# Longitudinal studies on cohesion in a military context – A systematic review

**DOI:** 10.1080/08995605.2022.2041995

**Published:** 2022-03-02

**Authors:** Maria Fors Brandebo, Marcus Börjesson, Hilmar Hilmarsson

**Affiliations:** Department of Security, Strategy and Leadership, Swedish Defence University, Karlstad, Sweden

**Keywords:** Cohesion, military, antecedents, outcomes, military groups

## Abstract

Cohesion is one of the most studied group phenomena and there is an agreement among scholars today that cohesion is a key contributor to team functioning and performance. A large body of research has shown that cohesion has several positive effects on psychological, social, and behavioral outcomes. Since research on cohesion has increased significantly in recent decades there is a need for an updated overview of research regarding antecedents and outcomes of cohesion in a military context. In this paper, a systematic literature review is conducted. The paper adheres to suggestions by scholars, relating the results in accordance with the dimensionality (i.e. social, task, or general) and organizational level of the construct (i.e. horizontal, vertical, or organizational) as well as focusing exclusively on studies with a longitudinal design. The paper highlights gaps in the literature and provides direction for future research.

**What is the public significance of this article?—**This paper reviews longitudinal studies on antecedents and outcomes of cohesion in a military context. Although the popularity of cohesion studies, there are surprisingly few longitudinal studies. The need for valid and reliable measures are highlighted as well as the need for including other dimensions than social and horizontal (peer) cohesion. There is also a need for studies on long-term effects of cohesion interventions.

## Introduction

A central part of work in the military is executed by individuals working together in groups, whether it is solving tasks in smaller group units (i.e. squad) or larger cooperative units (i.e. platoon). Therefore, knowledge regarding factors and processes that facilitate effective teamwork, as well as well-being and job-satisfaction among team members, is of great importance within the military research. Previous studies have shown that cohesion represents one such crucial group factor (e.g., Goodwin et al., 2018; Oliver et al., 1999). The purpose of the present study is to give an overview of antecedents and outcomes of group cohesion, specifically based on previous studies conducted in a military context and with a longitudinal design that have undergone a peer-review process.

Cohesion is one of the most studied group phenomena, and there is an agreement among scholars today that cohesion is a key contributor to team functioning and performance (Rosh et al., 2012). A large body of research has shown that cohesion has several positive effects on psychological, social, and behavioral outcomes. For example, previous research indicates relationships between cohesion and increased job and team satisfaction (Ahronson & Cameron, 2007), higher motivation (Gully et al., 1995), lower turnover (Griffith, 2002), reduced stress (Griffith & Vaitkus, 1999), increased well-being (Bliese & Halverson, 1996), enhanced team learning (Mullen & Copper, 1994) and group performance (Evans & Dion, 2012). Several reviews and meta-analyses have also been published that further underscore the positive effects of cohesion for team effectiveness (e.g., Beal et al., 2003; Chiocchio & Essiembre, 2009; Mullen & Copper, 1994; Salas et al., 2015). However, only one (Oliver et al., 1999) distinctively focuses on the military context. Oliver and colleagues examined 39 military studies and found a significant relationship between group cohesion and task performance. Two drawbacks with this study are that it does not apply a peer-review criterion, and that it was published 20 years ago. Although other literature reviews on cohesion include military studies, the specific characteristics of the military context (for example, unpredictable, potentially life-threatening, stressful) warrant a review of studies only conducted in this specific setting.

Despite this extensive amount of research, there is still a great deal of inconsistency in the literature regarding cohesion, and different opinions about how to operationalize and measure this construct (Severt & Estrada, 2015). Grossman et al. (2015) point to a number of different disagreements. For one thing, should it be considered a unidimensional or multidimensional construct? Based on their literature review, Grossman et al. (2015) state that cohesion should be seen as multidimensional, and that the two most relevant dimensions are task and social cohesion. According to Siebold (2007), social cohesion captures the emotional bonds of friendship, liking, caring, and closeness among group members, whereas task cohesion refers to the shared commitment of members to achieve a goal that requires the collective efforts of the group. These two dimensions of cohesion are also highlighted in one of the most frequently cited definitions of cohesion which describes cohesion as “a dynamic process that is reflected in the tendency for a group to stick together and remain united in the pursuit of its instrumental objectives and/or for the satisfaction of member affective needs” (Carron et al., 1998, p. 213).

Furthermore, cohesion has been studied on different levels within organizations. The most commonly studied type of cohesion is what has been termed horizontal cohesion which represents the perceptions of cohesion on the primary group level (Griffith, 1988). Another type of cohesion is vertical cohesion which refers to the degree group members identify with and positively relate to their leaders (Siebold & Kelly, 1988). Organizational cohesion is a third form of cohesion and captures the degree which group members identify and are attracted to the larger organization, such as the army (Siebold & Kelly, 1988).

Santoro and colleagues (Santoro et al., 2015) recognize the extensive support of cohesion being an important aspect of team effectiveness and group performance. At the same time, they are surprised by the relatively little knowledge there is about antecedents and relationships with other group processes and outcomes. Indeed, a dominant part of the research, reviews, and meta-analyses has exclusively focused on the cohesion-performance relationship while less is known about how one can build cohesion in a group (Casey-Campbell & Martens, 2009; Grossman et al., 2015; Kozlowski & Ilgen, 2006). Furthermore, Santoro et al. (2015) attribute the lack of knowledge partly to the fact that cross-sectional research designs and retrospective self-reports have predominated the way of studying cohesion. Thus, to gain more profound knowledge about the causal relationships between antecedents to and outcomes of cohesion, we should preferably base our understanding on longitudinal studies.

To summarize, the cohesion research literature suggests in general that cohesion should be viewed as a multidimensional and multilevel construct. Therefore, it is important to distinguish between different dimensions and levels to understand both the theoretical complexity of the construct as well as the practical implications. Furthermore, research studies lean heavily toward the cohesion–performance relationships. Hence, there seems to be a lack of clarity regarding antecedents and other relevant outcomes. Also, only one previous review has been done in the military context exclusively (i.e. Oliver et al., 1999), and it was published more than 20 years ago. Military groups are particularly interesting as they operate in areas with high complexity and they require high coordination among members to solve tasks. Previous research has shown that cohesion is especially relevant in contexts that demand strong interdependence (Beal et al., 2003; Chiocchio & Essiembre, 2009).

This brief review suggests that there is a need for an updated overview of the research regarding antecedents and outcomes of cohesion in a military context. Therefore, in this paper, a systematic literature review is conducted, with the purpose of identifying important antecedents and outcomes, relevant for military personnel. Furthermore, the paper adheres to suggestions by scholars, relating the results in accordance with the dimensionality (i.e. social, task, or general) and organizational level of the construct (i.e. horizontal, vertical, or organizational), as well as focusing exclusively on studies with a longitudinal design.

## Method – Literature review process

A systematic mixed studies review (Polit & Beck, 2012) with an integrated design (Sandelowski et al., 2006) was undertaken to integrate and synthesize findings from qualitative, quantitative, and mixed studies. The design was chosen to gain broader knowledge of cohesion as a phenomenon in military context. The literature review was based on the principles established by the Cochrane Collaboration (Higgins & Green, 2011) and the Preferred Reporting Items for Systematic Reviews and Meta-Analyses (PRISMA) guidelines (Moher et al., 2009). Our review was also methodologically inspired by the reviews of Kennedy et al. (2014) and Sansdalen et al. (2015).

We searched the following six databases in October 2019 and in January 2022 using identical searches: PsychINFO, Academic Search Elite, CINAHL, ERIC, PubMed, and Sociological abstracts (covering the whole time period of each data base). We limited our inclusion criteria to peer-review research articles, journal articles or dissertations, and also only English-medium articles. The searches were conducted using the descriptors “cohesion” AND (military OR army OR navy OR marines OR “air force” OR “special forces” OR reserve OR “coast guard” OR “national guard). The strategy also included a manual search of the reference lists in the studies selected from electronic search. The electronic database searches identified 1,459 records, with 88 additional records found in the reference lists. This yielded a total of 1,320 records after duplicates had been removed.

The selection of studies was conducted by applying a set of inclusion and exclusion criteria. The inclusion criteria were (1) longitudinal design, (2) the sample consisted of military personnel only, (3) the participants could be students, but they had to be in training for a military career. Groups including both military and civilian personnel did not qualify unless data were reported for each group, (4) the study had a measurement or description of cohesion as social-, task-, general, horizontal-, vertical- or organizational cohesion. The study reported a quantitative or qualitative relation between cohesion and an outcome or between cohesion and antecedents, and (5) studies can be quantitative, qualitative, mixed, case studies, or experimental.

The exclusion criteria were as follows: (1) Development of measurement scales, (2) review studies or Meta-analysis studies, (3) no clear definition of cohesion or definition of measurement of cohesion, and (4) cohesion as pride, cohesion as social groups, choosing other team members or cohesion in therapeutic settings.

**Figure 1.**
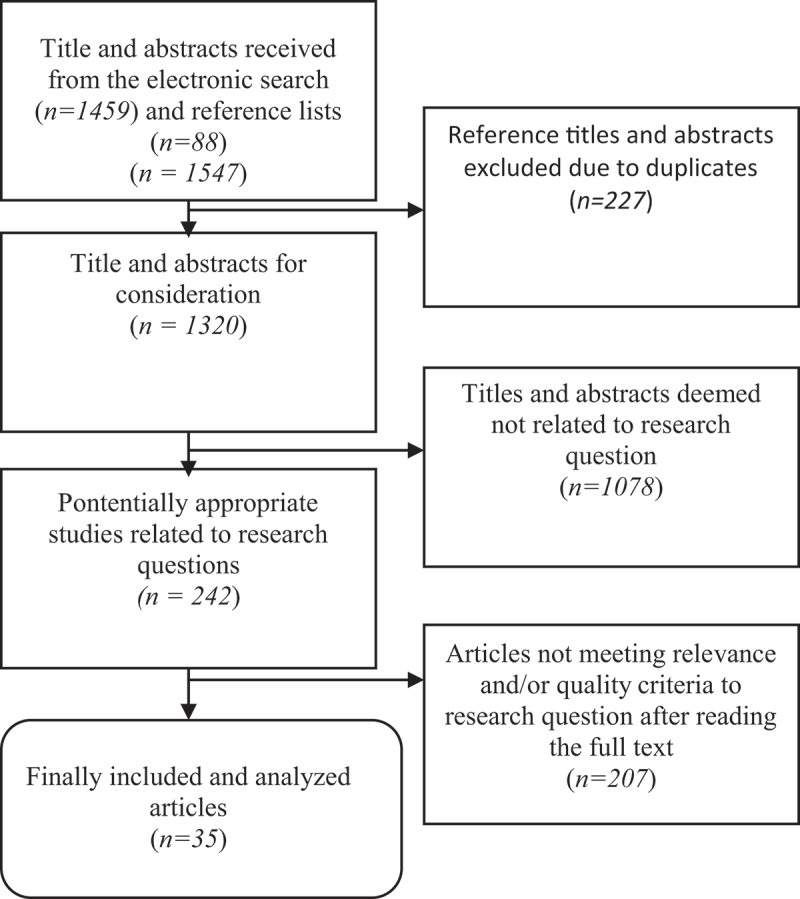
Search results.

Titles and abstracts were screened regarding the inclusion and exclusion criteria, and if study aims were met. As a result, 1078 records were rejected, leaving 242 articles. These were assessed in full text and an additional 207 were rejected not meeting inclusion criteria. This process rendered 35 papers for inclusion (see, Figure 1).

## Results – A review of longitudinal cohesion literature in a military context

### Antecedents to cohesion

Our review identified 15 empirical studies that have investigated antecedents to cohesion in a military context. Ten of these were conducted in training settings while five took place during service/deployment. A clear majority, 13 studies, studied horizontal (unit) cohesion. Only two studies addressed vertical cohesion (Orme & Kehoe, 2019; Vaitkus & Griffith, 1990) and one of these also included organizational cohesion (Orme & Kehoe, 2019). Two studies combined items related to both horizontal and vertical cohesion (Anderson et al., 2019; Jones et al., 2019).

Six of the studies have created their own measures for the specific study (Anderson et al., 2019; Bartone & Adler, 1999; Hadid et al., 2008; Johnston et al., 2019; Vogelaar & Kuipers, 1997). Three studies refers to the measurement instrument created by Siebold and Kelly (2002, 2019, 2019, 2015) and three have used a measure (or items) developed by Podsakoff and MacKenzie (2015, 2013, 1994, 2016).

Our literature review identified social cohesion as the most studied dimension of cohesion. Nine studies measure social cohesion, one measures task cohesion (Arthur & Hardy, 2014), while one study combines measures of both social and task cohesion in the same measurement (Anderson et al., 2019). One study gave no detailed description of the measure, but the sample item included indicates they measured social cohesion (Eathough et al., 2015). One study uses a one-item measure (what is the level of cohesion in your unit at this time, Bartone & Adler, 1999).

#### Individual antecedents to cohesion

This literature review has identified nine studies that have examined individual-related antecedents to cohesion. Three of these investigates social cohesion and gender. Two of the studies did not find any differences between men and women (Horizontal cohesion, Hadid et al., 2008; Horizontal, vertical and organizational cohesion; Orme & Kehoe, 2019) although Hadid et al. (2008) discovered that males seemed to prefer males as team members and Orme and Kehoe (2019) saw a slight decline in vertical cohesion for women when the proportion of women in the unit increased. Kline et al. (2013) revealed that women were more likely to rate cohesion lower than men which the authors suggests is related to sexual harassment and occupational bias.

Two studies examined sociodemographic variables and cohesion. Bartone and Adler (1999) did not specify social or task cohesion, but used a general term instead. The results from the study show that late in deployment, military police, physicians, and communication workers rated cohesion highest, while administrative personnel and operating room staff rated cohesion lowest. Volunteers rated cohesion higher compared to non-volunteers. The authors did not find any racial differences. Anderson et al. (2019) discovered that males, individuals with shorter tenure, and soldiers with prior deployment rated cohesion higher. Ratings of cohesion also increased with age. In this study cohesion was measured with a factor combining both social and task cohesion as well as horizontal and vertical cohesion.

Although mental health is one of the most studied phenomena in relation to cohesion in military contexts (see, for example, Reed-Fitzke & Lucier-Greer, 2019), only one longitudinal study has examined mental health as an antecedent to social and task horizontal cohesion. When adjusting for baseline mental health, cohesion did not remain significantly associated with mental health (Thomassen et al., 2015). The authors also included ratings of hardiness, and for individuals scoring low on hardiness, higher levels of cohesion contributed to lower levels of mental health complaints.

Bartone et al. (2002) compared familiar teams (created one year before) with unfamiliar teams and discovered that familiar teams rated social, horizontal cohesion higher than did unfamiliar teams. Vaitkus and Griffith (1990) studied unit replacement units (UR) and individual replacement units (IR) and found that six months after the first measurement occasion UR rated both social, vertical cohesion, as well as social, horizontal cohesion higher than IR. Six months after the second-measurement occasion the only remaining difference was related to social, horizontal cohesion. Bartone and Adler (1999) found that, mid-deployment, the individual’s satisfaction with the role of the Joint Task Force predicted higher cohesion.

#### Group-related antecedents

Only one study has addressed group-related antecedents to cohesion. Bartone and Adler (1999) found that pre, mid- and late deployment the individual’s ratings of the unit’s ability to perform the mission predicted cohesion.

#### Organizational-related antecedents

This literature review discovered only one longitudinal study that examined organization-related antecedents to cohesion. The individual’s ratings of confidence in equipment as well as confidence that the family was taken care of predicted cohesion in late deployment but not pre- or mid deployment (Bartone & Adler, 1999).

#### Leadership-related antecedents

In this literature review, six studies were identified that investigated leadership-related antecedents to cohesion. Although measuring different aspects of leadership, such as motivating, showing respect, and being tough (Foran & Adler, 2013), concerned leadership (Bartone & Adler, 1999; Vaitkus & Griffith, 1990), confidence in leaders (Bartone & Adler, 1999) or transformational leadership (Arthur & Hardy, 2014) all but one study point to leadership being important for the development of cohesion. Vogelaar and Kuipers (1997) studied how the perceived effectiveness of four commander levels predicted social, horizontal cohesion. Their results showed no significant associations except for the effectiveness of the deputy group commander measured 7–8 months into service and cohesion 11 months into service.

#### Situation-related antecedents

One longitudinal study was found in the literature review that was related to situation-related antecedents to cohesion. Bartone et al. (2002) discovered that a combined effect of being familiar in the unit and experiencing, as a group, a stressful event led to higher social, horizontal cohesion.

#### Interventions

Four studies related to interventions were discovered, and they are all carried out within the last five years. Most of them studied social and horizontal cohesion. Common for the studies is that they take place over a relatively short time interval extending from a few hours (ropes course challenge: Eathough et al., 2015), a few days (Team training: Johnston et al., 2019) to a few months (Resilience vs. military history training: Adler et al., 2015; Military resilience intervention: Jones et al., 2019). The results from these studies indicate no larger significant differences between the different groups in the studies except for the results from a study examining horizontal cohesion before and after a ropes course challenge that showed significant changes (Eathough et al., 2015). None of the studies address long-term effects.

### Outcomes of cohesion

The review identified 18 longitudinal and peer-reviewed articles that have examined outcomes of cohesion in a military context. Four of these were conducted in training settings and 14 were conducted during service/deployment. All of the articles studied horizontal (unit) cohesion. Six of the articles (Anderson et al., 2019; Breslau et al., 2016; Bury, 2018; Campbell-Sills et al., 2022; Orme & Kehoe, 2020; Steenkamp et al., 2014) also included items reflecting vertical cohesion and three included items of organizational cohesion (Breslau et al., 2016; Bury, 2018; Orme & Kehoe, 2020). However, a majority of the articles that included different types and items of cohesion used a combined index (unit cohesion) in the statistical analyses. Thus, they did not examine the separate effect of horizontal, vertical, and organizational cohesion, respectively. The exceptions are Campbell-Sills et al. (2022), Orme and Kehoe (2020), and Steenkamp et al. (2014), who examined outcomes for both horizontal and vertical cohesion.

Three of the studies have used their own measures for the specific study (Anderson et al., 2019; Gilbar et al., 2010; Maguen & Litz, 2006). Four studies refer to the measurement instrument created by King et al., (2006; Breslau et al., 2016; Han et al., 2014; Kline et al., 2013; Polusny et al., 2011), three studies use measures (or items) from Podsakoff and MacKenzie (2005, 1994, 2014, 2016), three studies refer to Siebold and Kelly (2018, 2020, 1988, 2015), one study uses measures from Carron et al. (1985; Börjesson et al., 2011), one study refers to Manning and Ingraham (1983; Steenkamp et al., 2014), one study refers to Mangelsdorff and Moses (1993, 2013), one study refers to Seers (1989: Jordan et al., 2002), one study uses measures from Wright et al. (2017, 2009) and one study (Campbell-Sills et al., 2022) uses items from the PDDS panel survey (Kessler et al., 2013).

Six of the articles in the review measure social cohesion (Breslau et al., 2016; Han et al., 2014; Jordan et al., 2002; Kline et al., 2013; Polusny et al., 2011; Smith et al., 2013). Nine articles combine measures of both social and task cohesion in the same measurement (Anderson et al., 2019; Britt & Dawson, 2005; Bury, 2018; Campbell-Sills et al., 2022; McAndrew et al., 2017; Orme & Kehoe, 2020; Steenkamp et al., 2014; Thomassen et al., 2015; Williams et al., 2016). Only one article (Börjesson et al., 2011) measures and analyses social and task cohesion separately. Furthermore, two studies uses a single item as a measurement of cohesion (Gilbar et al., 2010; Maguen & Litz, 2006).

#### Individual outcomes

The review revealed that 16 of the 18 identified longitudinal studies have examined individual-related outcomes of cohesion. The majority (11 articles) of these articles studies relationships between cohesion and mental health issues, especially psychological stress/symptoms. Three articles examine relationships between cohesion and well-being factors, and an additional five articles study the association between cohesion and work-related behaviors or attitudes.

Results from the articles show that stronger pre-deployment cohesion is related to less risk of developing post-deployment PTSD (Anderson et al., 2019; Breslau et al., 2016; Han et al., 2014; Kline et al., 2013; Polusny et al., 2011), depression (Anderson et al., 2019; Breslau et al., 2016), anxiety (Anderson et al., 2019), distress (Gilbar et al., 2010), alcohol or substance disorder (Anderson et al., 2019) and negative mental health (Thomassen et al., 2015). Furthermore, Steenkamp et al. (2014) analyzed results from a multi-wave longitudinal field cohort study of active duty ground-combat Marines who deployed to Iraq or Afghanistan between 2008 and 2012. The result showed that both stronger horizontal and vertical cohesion was associated with less perceived stigma of seeking mental health care. Similarly, Campbell-Sills et al. (2022) analyzed data from the Pre/Post-Deployment Study (PPDS), conducted among U.S. soldiers deployed to Afghanistan. The result showed that, at the individual-level, horizontal cohesion had a buffering effect for post-deployment (3 months after deployment) PTSD and depressive symptoms, while a buffering effect of vertical cohesion was found for PTSD symptoms only. At the unit-level horizontal cohesion had a buffering effect for PTSD, depressive symptoms as well as suicidal ideation. However, in regard to alcohol use, a study by Breslau et al. (2016) show a contradictory result, showing that higher unit cohesion was related to a higher likelihood of alcohol misuse. The authors suggest that it is likely that an important marker for unit cohesion is drinking together.

Two articles also examine relations between cohesion and psychological stress symptoms during training. Smith et al. (2013) studies Marine recruits at the beginning and end of a highly stressful 13-week training program and found that cohesion acted as a buffer, weakening the association between the stressfulness of the training and Posttraumatic Stress Symptomatology (PTSS). In a study on soldiers that took part in a 10-week Basic Combat Training (BCT), it was found that stronger cohesion was associated with decreases in psychological distress, sleep problems, and tolerance of BCT stressors (Williams et al., 2016).

Three articles have found relationships between cohesion, psychological well-being and resilience factors. Britt and Dawson (2005) studied U.S. soldiers stationed in Europe and found that unit cohesion was negatively associated with a well-being factor, as stronger cohesion was related to increased Work-Family conflict. However, this relationship was only true when soldiers also felt a low sense of job significance. Studying Army National Guard and reserve personnel after deployment, unit cohesion was associated with a reduction in avoidant coping. The reduction, in turn, mediated the relationship between unit cohesion and improvement in mental health functions (McAndrew et al., 2017). Furthermore, Williams et al. (2016) study on soldiers in a 10-week BCT (mentioned above) also showed that stronger cohesion was associated with an increase in resilience, confidence in managing stress reactions, and positive states of mind.

In regard to work-related factors, Börjesson et al. (2011) conducted a study on Swedish conscripts during their compulsory military training. They found that conscripts who reported higher horizontal task cohesion felt to a lesser extent that unnecessary risk was taken during their training. The authors argue that cohesion has a positive effect on the subjective sense of feeling in control in risk situations. In a study (Maguen & Litz, 2006) of U.S. military peacekeepers deployed in Kosovo, it was found that unit cohesion predicted pre-deployment morale while post-deployment unit cohesion predicted post-deployment morale. Higher unit cohesion has also been related to a lower likelihood of violations of the uniformed conduct of military justice (Breslau et al., 2016). Williams et al. (2016) study on BCT also revealed that cohesion was indirectly associated with physical performance and Basic Rifle Marksmanship through cohesion-related improvement in psychological distress, resilience and confidence in managing stress reactions and tolerance of BCT stressors. Finally, a study (Orme & Kehoe, 2020) on Australian Army soldiers, during their specialty training, showed that instructor ratings of horizontal cohesion had a positive association with section-level performance grades.

#### Group-related outcomes

Only two longitudinal studies have examined group-related outcomes in a military context. Jordan et al. (2002) studied military officers working in self-managed teams during a 5-week period, and found that social cohesion correlated with mental task performance, physical task performance, and commander ratings of team performance. In a study on British Army Reserve logistics soldiers, Bury (2018) found that units with higher cohesion reported higher morale and military readiness.

## Discussion

The aim of this systematic review was to identify important antecedents and outcomes, relevant for military personnel by reviewing longitudinal peer-review papers conducted in military settings. Based on our literature review a number of key observations are described below. First, we identified a large number of studies on cohesion in a military context of which 35 met the inclusion criteria. This is a relatively low number of studies in light of that cohesion is one of the most studied group phenomenon (Severt & Estrada, 2015).

Second, regarding antecedents to cohesion very few studies seem to be conducted in real-life settings since most of them are carried out during training or exercises. Although a large part of the military profession consists of preparation for operations, there is a lack of knowledge of which antecedents can contribute to cohesion during service. Especially since previous studies show that cohesion increases during training conditions (see, for example, Bartone et al., 2002). Interesting enough, the opposite is in evidence regarding outcomes. Of the studies identified related to outcomes of cohesion, a majority (14 out of 18) are conducted during service/deployment. In this regard, we know little about cohesion outcomes during training and exercises.

Third, our literature review shows that the identified studies use many different construct measures that are not comparable. This means that there is a lack of valid and reliable measures. If this field of research should advance, researchers need to develop and use the same measures. Another limitation in the identified studies is that most of them have only studied social cohesion. Grossman et al. (2015) emphasized that cohesion should be divided into and studied based on the dimensions social and task-related cohesion. The distinction between these two dimensions of cohesion is significant, since they have been demonstrated to be differently related to various outcomes (e.g., Beal et al., 2003; Carless & De Paola, 2000; Dion, 2000; Mullen & Copper, 1994). Further, most studies have only investigated horizontal cohesion and have neglected vertical and organizational cohesion. Scholars have pointed to the importance of studying cohesion across hierarchical levels as cohesion could exist at different levels within a hierarchy (Griffith, 1988; Severt & Estrada, 2015), for example, ranging from the individual, unit, leader, and organizational level within the military (Salo, 2011). Hence, we recommend that further studies within the military field pay closer attention to and address the multidimensionality (i.e. social and task cohesion) multi-level concepts of cohesion.

Moving on to our fourth key observation, most of the studies in this literature review use self-reported data. Thus, the relationships between cohesion, antecedents, and outcomes identified in this study may partly be attributable to common method variance (Podsakoff et al., 2012) and by social desirability (Crowne & Marlowe, 1960). This is a known problem for researchers that it is difficult to obtain data other than self-reported data. Nevertheless, challenges for further studies are to identify objective variables as well as to use different methodological approaches, other than survey studies.

The next observation is related to long-term effects of interventions and actions to increase cohesion. Few studies have investigated the long-term effects of these kind of efforts. For example, it could be only a few hours (Eathough et al., 2015) or a few days (Johnston et al., 2019) between measurement points. The longest time intervals were a few months (Adler et al., 2015; Jones et al., 2019). This shows that we still do not have sufficient knowledge on what causes cohesion to develop or maintain at a high level over time.

Our sixth and final observation is related to what seems to be favorable for developing and maintaining cohesion. Starting with antecedents to cohesion, the results points to leadership being an important aspect. Due to a small number of studies using different leadership measures it is difficult to draw any conclusions on what kind of leadership behaviors are more or less effective when it comes to increasing and maintaining cohesion. However, the results point to motivating, showing respect and consideration and transformational leadership as important. Surprisingly, not one of the identified studies has examined destructive leadership in relation to cohesion. This is unexpected since the research field of destructive leadership has increased in recent decades and researchers have studied this association in the civilian context although mostly related to passive forms of destructive leadership (Laissez-faire) (see, for example, Nielsen, 2013). Regarding outcomes of cohesion it is less surprising that most of the studies are related to the association between cohesion and mental health. Mental health in the military context is well researched (see, for example, Pietrzak et al., 2012) and this literature review also confirms a significant association. In civilian contexts, many researchers have shown interest for the association between cohesion and work-related outcomes, such as job satisfaction and intention to remain in the organization. Our literature review shows that there are very few longitudinal studies with this focus in military contexts. It should be important to gain more knowledge on these aspects since many armed forces have all-volunteer forces, making it important to retain their personnel. For example, Sweden has experienced that it has been relatively easy to recruit all-volunteer soldiers and sailors, but much more difficult to retain them (Jonsson, 2009). Knowledge about how to increase and maintain cohesion could lead to higher job satisfaction and lower turnover.

### Summary and agenda for future research

This literature review identified a surprisingly small number of longitudinal studies focusing on antecedents to and outcomes of cohesion. It is apparent that this area of research needs and deserves greater attention. In future research, valid and reliable measures need to be used, and studies should include dimensions other than only social and horizontal cohesion. It is also important to study long-term effects of cohesion interventions in order to gain information regarding which measures are worthwhile. Leadership seems to be a significant antecedent to cohesion, although more studies are needed. Research that investigates what kind of behavior of leaders has the most favorable and unfavorable effects on different types of cohesion is important for individuals, teams, and organizations. This knowledge should be most important in leadership intervention and education.

## Data Availability

The authors confirm that the data supporting the findings of this study are available within the article.
